# Silencing a Multifunctional microRNA Is Beneficial for Stroke Recovery

**DOI:** 10.3389/fnmol.2018.00058

**Published:** 2018-02-23

**Authors:** Tamara Roitbak

**Affiliations:** Department of Neurosurgery, Health Sciences Center, University of New Mexico, Albuquerque, NM, United States

**Keywords:** microRNA, miR-155, dMCAO, post-stroke inflammation, cerebral blood flow, functional recovery

## Abstract

Stroke-induced endothelial cell injury leads to destruction of cerebral microvasculature and significant damage to the brain tissue. A subacute phase of cerebral ischemia is associated with regeneration involving the activation of vascular remodeling, neuroplasticity, neurogenesis, and neuroinflammation processes. Effective restoration and improvement of blood supply to the damaged brain tissue offers a potential therapy for stroke. microRNAs (miRNAs) are recently identified small RNA molecules that regulate gene expression and significantly influence the essential cellular processes associated with brain repair following stroke. A number of specific miRNAs are implicated in regulating the development and propagation of the ischemic tissue damage as well as in mediating post-stroke regeneration. In this review, I discuss the functions of the miRNA miR-155 and the effect of its *in vivo* inhibition on brain recovery following experimental cerebral ischemia. The article introduces new and unexplored approach to cerebral regeneration: regulation of brain tissue repair through a direct modulation of specific miRNA activity.

## Introduction

Stroke is a major public health problem in the United States, with 795,000 stroke survivors each year (610,000 of these are first attacks). According to the global stroke burden assessment, in 2013 there were more than 25 million stroke patients worldwide, with ∼10.3 million having a first stroke. Ischemic strokes account for up to 71% of all stroke cases and 51% of all stroke-related deaths worldwide, and they are currently among the top leading causes of serious, long-term disability (Feigin et al., 2016). Stroke-associated ischemic damage involves cellular bioenergetic failure, excitotoxicity, oxidative stress, blood–brain barrier dysfunction, microvascular injury, post-ischemic inflammation, and, ultimately, the death of neurons, glia, and endothelial cells. The endothelial cell damage leads to a significant microvascular injury that directly contributes to cerebral tissue damage via increased endothelial cell permeability, matrix degradation, and the loss of cerebrovascular autoregulation (Brouns and De Deyn, 2009; Xing et al., 2012). The ischemic cascade leads to the development of the infarct core, which is represented by non-viable brain tissue. A hypoxic, but still viable, peri-infarct area surrounding the core region is a subject of intensive investigation, with a focus on neuroprotective and pro-regenerative treatments for preservation of salvageable brain tissue. The subacute phase of ischemia is accompanied by the active regeneration process, including post-ischemic angiogenesis and vasculogenesis in the ischemic boundary zone, which enhances oxygen and nutrient supply to the affected tissue (Ding et al., 2008; Beck and Plate, 2009). Stroke induces a potent neurogenic response and a massive migration of neural progenitors into the lesion area, which may substantially contribute to recovery and repair processes (Arvidsson et al., 2002; Kernie and Parent, 2010). In addition, cerebral ischemia triggers neuronal plasticity reflected in formation of new structural and functional connections between the neurons from the area adjacent to the infarcted tissue and the surrounding “healthy” brain tissue of the same hemisphere (Carmichael et al., 2017). Post-ischemic inflammatory response is an integral part of both brain damage and recovery. Post-ischemic elevation of cytokines is associated with recruitment of neutrophils, lymphocytes, and monocytes and activation of resident microglia, astrocytes, and endothelial cells. Activation of microglia and astrocytes leads to the additional release of pro-inflammatory factors (Liguz-Lecznar and Kossut, 2013). Lower levels of pro-inflammatory cytokines and higher expression of anti-inflammatory cytokines are associated with lower infarct size and a better clinical outcome (Perera et al., 2006; Lakhan et al., 2009). However, there is controversy surrounding the dual role of neuroinflammation in tissue damage and recovery: the post-stroke inflammation events contribute to brain injury but, on the other hand, could participate in tissue remodeling and recovery following brain damage. All of the described functionally-linked regeneration processes (including vascular remodeling, neural stem cell activation, neuronal plasticity, and neuroinflammation) reflect crosstalk between the components of the injured brain tissue. Molecular mechanisms of this complex repair process are extensively studied in search of possible targets for therapeutic intervention.

### Strategies to Improve a Post-stroke Recovery

Despite the recent progress in post-stroke survival, therapeutic approaches directed toward recovery remain limited. Traditional approaches utilizing tissue-plasminogen activator (rt-PA) for thrombolysis are associated with time limitations (a 3–4 h therapeutic window) and possible complications (Lakhan et al., 2009). Neurorestorative processes may be enhanced by improving angiogenesis, neuroplasticity, and neurogenesis. Proposed approaches for vascular repair include the injection of angiogenesis-promoting compounds (Navaratna et al., 2009) or the transplantation of the endothelial progenitors (Fan et al., 2010; Bai et al., 2015) or neural stem/progenitor cells (Roitbak et al., 2008). Stem cell-based therapy for stroke and current pharmacological and tissue engineering approaches are discussed in detail in most recent reviews (Carmichael, 2016; Venkat et al., 2017).

All of the described therapeutic approaches are associated with certain limitations and complications, such as the increased vascular permeability and edema following VEGF treatment or possible transplant rejection, tumor formation, and infection associated with progenitor cell transplantation. An emerging and yet unexplored approach for treatment is based on the epigenetic processes associated with stroke (Pearce, 2011; Kassis et al., 2017). Among the epigenetic mechanisms that regulate stroke progression and recovery are the signaling pathways mediated by recently identified short non-coding RNAs called microRNAs.

## microRNAs

miRNAs are a diverse class of highly conserved small RNA molecules that function as critical regulators of gene expression and are able to greatly influence cell development, differentiation, proliferation, and apoptosis (Kato and Slack, 2008). It is estimated that a large number of encoded genes are regulated by these single-stranded non-coding short (18–24 nucleotides long) RNAs. The miRNAs bind to their mRNA target at complementary sequences and downregulate gene expression by inhibiting the mRNA translation into proteins or by inducing mRNA degradation (Sun et al., 2010). The miRNAs control post-transcriptional gene expression in many tissues, including the brain (Ludwig et al., 2016). miRNA profiles have been characterized in the cerebral vasculature, neurons, astrocytes, and microglia (Kosik, 2006; Butovsky et al., 2014; Lopez-Ramirez et al., 2014; Rao et al., 2016).

Recent findings demonstrate that miRNAs orchestrate a variety of signaling pathways involved in stroke progression and post-stroke recovery, including neurogenesis (Zhao et al., 2008; Shi et al., 2010), endothelial cell morphogenesis (Poliseno et al., 2006; Suarez et al., 2007), and neuroinflammation (Gaudet et al., 2017). Multiple miRNAs have been found to play a profound role in stroke progression. The role of the miRNAs and the associated molecular mechanisms are discussed in detail in several excellent reviews (Lim et al., 2010; Vemuganti, 2010; Khoshnam et al., 2017; Li et al., 2017). Significant changes of miRNA profiles in the brain tissue and blood were detected at different times after the experimental ischemia in rodents (Liu et al., 2010). Numerous studies have reported a significant deregulation of specific circulating miRNAs in stroke patients (Jickling et al., 2014; Sepramaniam et al., 2014). These distinctive temporal changes are now regarded as indicators of the risk, occurrence, severity, and prognosis of stroke and have prompted an extensive search for specific peripheral miRNAs as biomarkers of the disease (Vijayan and Reddy, 2016). Another approach constitutes targeted regulation of the specific microRNA activity to stimulate a recovery process after stroke.

### *In Vivo* Regulation of miRNAs

Systemic or local administration of the miRNA inhibitors and mimics are utilized to regulate the activity of specific miRNAs in various experimental animal models, including brain tumor (Teplyuk et al., 2016), breast cancer (Zhao et al., 2013), pancreatic cancer (Gibori et al., 2018), and pulmonary hypertension (McLendon et al., 2015) disease models. The first steps are performed to apply this technology to humans, such as subcutaneous delivery of anti-miR-122 in chronic hepatitis C patients (Stelma et al., 2017; van der Ree et al., 2017). This method could provide a future therapeutic potential in treatment of various diseases (Linnstaedt et al., 2010; Zhang et al., 2013). *In vivo* regulation of miRNAs in the animal models of stroke has been achieved by direct intracerebroventricular injections of specific antagomirs for miRNAs miR-493 and miR-23a-3p (Zhao et al., 2013; Li et al., 2016). Our recent investigations involving the *in vivo* inhibition of miRNA miR-155 introduced a novel approach to cerebral regeneration after stroke: regulation of post-stroke recovery via systemic injection of specific miRNA inhibitor.

### miR-155 Functions

miR-155 is a multifunctional miRNA implicated in regulating various physiological and pathological processes such as hematopoietic lineage differentiation, immunity, inflammation, cancer, and cardiovascular diseases (Faraoni et al., 2009; O’Connell et al., 2010). miR-155 is specifically expressed in hematopoietic cells and cells involved in vascular remodeling, including B-cells, T-cells, monocytes, and granulocytes as well as endothelial cells and smooth muscle cells (Landgraf et al., 2007; Sun et al., 2012). Among other functions, miR-155 is involved in regulating the endothelial and vascular function (Sun et al., 2012; Weber et al., 2014). Downregulation of this miRNA is accompanied by reduced inflammation and improved regeneration processes (Kurowska-Stolarska et al., 2011; Murugaiyan et al., 2011; van Solingen et al., 2014). In addition to these multiple functions, miR-155 is involved in progression of multiple CNS disorders and pathological conditions. Some of the recent studies describing these miR-155 functions are summarized in **Table 1**.

**Table 1:** Involvement of miR-155 in CNS disorders and pathological conditions.

Amyotrophic lateral sclerosis (ALS)	Increased expression in CNS tissue from ALS patients and in mouse model of ALS (SOD1 mice). Inhibition prolongs survival and ameliorates disease in SOD1 mice (Koval et al., 2013; Butovsky et al., 2015).
Epilepsy	Regulates glutamate uptake capacity of astrocytes in epilepsy (Gao et al., 2017). Significantly upregulated in the hippocampal tissues of children with Medial temporal lobe epilepsy (Ashhab et al., 2013).
Stroke	Expression significantly affected by cerebral ischemia in rodents (Liu et al., 2010). Increased expression in rat model of stroke; inhibition supports post-stroke recovery (Caballero-Garrido et al., 2015; Xing et al., 2016).
Experimental autoimmune encephalomyelitis (EAE)	Reduced severity of EAE in miR-155 KO mice (Murugaiyan et al., 2011). Knockdown results in reduction of Th1 and Th17 cells and mild EAE (Zhang et al., 2014).
Spinal cord injury	Improved locomotor recovery in miR-155 KO mice (Yi et al., 2015; Gaudet et al., 2017).
Multiple sclerosis (MS)	Upregulated in blood and brain samples of MS patients (Lopez-Ramirez et al., 2014; Zhang et al., 2014).
Alzheimer’s disease (AD)	Upregulation contributes to neuroinflammation in transgenic mouse model of AD (Guedes et al., 2014). Regulates T-lymphocyte activation and function in AD (Song and Lee, 2015).
Parkinson’s disease (PD)	Increased expression in mouse model of PD; mediates inflammatory responses (Thome et al., 2016).
Brain tumor	Promotes glioma cell proliferation and contributes to glioma progression (Zhou et al., 2013; Yan et al., 2015); Increased expression in glioma tissue is associated with poor prognosis in patients (Sun et al., 2012). Upregulated in glioblastoma and promotes tumor growth (D’Urso et al., 2012).

## Effect of Systemic miR-155 Inhibition After the Experimental Cerebral Ischemia

Our *in vitro* studies identified miR-155 as a potential regulator of the endothelial morphogenesis: specific miR-155 antisense inhibitors supported capillary-like tube formation by the mouse brain endothelial cells (Roitbak et al., 2011). We hypothesized that the inhibition of miR-155 after the experimental ischemia could support cerebral vasculature and improve vascular function. Intravenous injections of a specific anti-miR-155 inhibitor, initiated at 48 h after mouse distal middle cerebral artery occlusion (dMCAO), resulted in ∼50% downregulation of miR-155 in the injured hemisphere of the mouse brain. *In vivo* two-photon laser scanning microscopy imaging and quantification of red blood cell (RBC) flow velocity detected a significantly improved blood flow in the peri-infarct area of the miR-155 inhibitor-injected mice during the first 2 weeks after dMCAO (Caballero-Garrido et al., 2015). These animals also demonstrated an improved vascular integrity and well-preserved capillary tight junctions (TJs). An assessment of the brain tissue damage using MRI and electron microscopy (EM) demonstrated that at 3 weeks after stroke there was a significant (34%) reduction of the infarct size and a significant decrease in neuronal damage in miR-155 inhibitor-injected animals, as compared to the control group. Improved TJ integrity in the inhibitor-injected animals was accompanied by the increased expression of major TJ protein ZO-1 and miR-155 target protein Rheb (Caballero-Garrido et al., 2015).

miR-155 inhibition after dMCAO significantly altered the time course and the expression levels of the major cytokines (including IL-10, IL-4, IL-6, MIP-1α, IL-5, and IL-17) as well as considerably modified the microglia/macrophage phenotype in the peri-infarct area of stroke. Electron microscopy-based quantification detected a decreased number of phagocytically active peri-vascular microglia/macrophages (M/Ms) in these animals (Pena-Philippides et al., 2016).

The assessment of sensorimotor deficits (bilateral asymmetry/adhesive removal test) and gait/locomotion recovery (CatWalk system), as well as the weight-gain evaluation, indicated that the inhibitor-injected animals regained their sensorimotor deficits and recovered faster than controls (Caballero-Garrido et al., 2015). Thus, miR-155-inhibition-induced support of the cerebral vasculature, preservation of brain tissue, and the modified course of post-stroke neuroinflammation reflected in more efficient functional recovery of the inhibitor-injected animals. To our knowledge, this is among first reports describing the efficacy of intravenous antagomir injections performed during the sub-acute phase of the experimental stroke. Another study involving intravenous anti-miR-181 demonstrated improved animal recovery in mouse transient focal ischemia model (Xu et al., 2015). Experiments utilizing miR-155 inhibition in the rat model of cerebral ischemia supported our studies and demonstrated the efficacy of anti-miR-155 treatment in rats (Xing et al., 2016). In this study, the intracerebroventricular injections of anti-miR-155 performed at 24 h before the MCAO resulted in reduced infarct size and improved recovery. Similar to our findings, this effect was associated with the activation of miR-155 target Rheb (Xing et al., 2016).

## Molecular Mechanisms of miR-155 Inhibition-Induced Support of Post-Stroke Recovery

**FIGURE 1:**
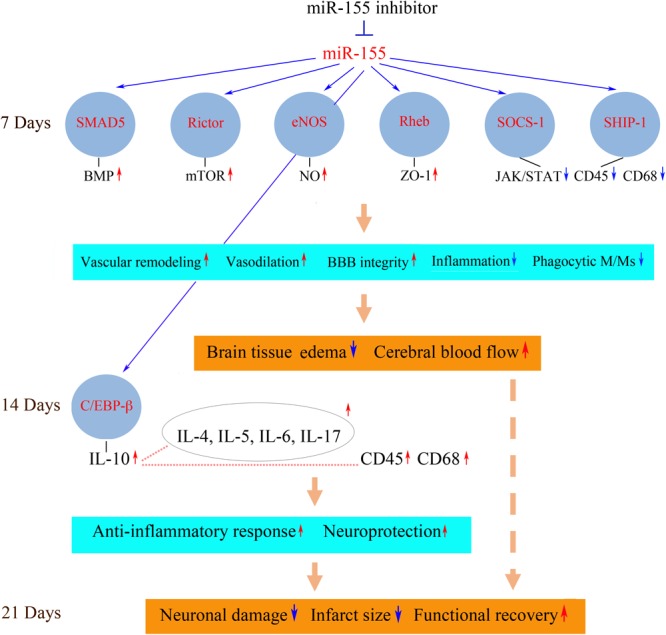
Possible molecular mechanisms of miR-155 inhibition-mediated support of post-stroke recovery. Possible molecular mechanisms mediating neurorestorative processes could be associated with the activation (red arrows) or repression (blue arrows) of miR-155-regulated signaling pathways. Downregulation of miR-155 results in the increased expression of miR-155 target proteins SMAD5, Rictor, eNOS, and Rheb at 7 days after dMCAO. These signaling molecules activate molecular pathways including BMP, mTOR, NO, and Akt/ZO-1, which strengthen the barrier function of the microvascular TJs. Upregulated miR-155 targets SOCS-1 and SHIP-1 suppress JAK/STAT-mediated cytokine signaling, which leads to a decreased number of CD45/CD68-expressing M/Ms and reduced inflammation. As a result, miR-155 inhibitor-induced support of BBB integrity leads to the reduction of brain edema and restoration blood flow in the peri-infarct area of stroke. At 14 days after dMCAO, there is an increased expression of the neuroprotective cytokines, including a major anti-inflammatory cytokine IL-10. IL-10 (possibly activated by miR-155 target C/EBP-β) could upregulate (dotted lines) other cytokines as well as M/M surface antigen CD45. IL-10-triggered neuroprotective response and delayed activation of phagocytic M/Ms facilitate the brain recovery process. The neuroprotective processes occurring at 7 and 14 days prevent delayed neuronal death in the peri-infarct area at 21 days after the experimental ischemia. Reduced brain infarct is accompanied by improved functional recovery. SMAD5, mothers against decapentaplegic homolog 5; eNOS, endothelial nitric oxide synthase; Rheb, Ras homolog enriched in brain; SOCS-1, suppressor of cytokine signaling molecule 1; SHIP-1, Src homology 2-containing inositol phosphatase 1; BMP, bone morphogenetic protein; mTOR, mammalian target of rapamycin; NO, nitric oxide; ZO-1, zonula occludens-1; JAK/STAT, Janus kinase/signal transducers and activators of transcription; CD45, protein tyrosine phosphatase, receptor type C/cluster of differentiation antigen 45; CD68, cluster of differentiation antigen 68; C/EBP-β, CCAAT/enhancer binding protein beta; Ccl12, chemokine ligand 12; CXCL3, chemokine (C-X-C motif) ligand 3; IL, interleukin. The diagram is adopted from Caballero-Garrido et al. (2015) in accordance with the Journal of Neuroscience Permissions Policy, with slight modifications based on the results published in Pena-Philippides et al. (2016).

Possible mechanisms and consequences associated with *in vivo* inhibition of miR-155 after stroke are summarized in **Figure 1**. The improved blood supply to the peri-infarct area of the injured hemisphere detected in the inhibitor-injected animals could be mainly attributed to miR-155 inhibition-induced preservation of the endothelial TJs and thus BBB integrity. Based on the analyses, strengthening of TJs could be mediated by miR-155 inhibition-induced stabilization of ZO-1, a regulatory scaffolding protein critical for the TJ assembly and signal transduction (Antonetti et al., 1999; Harhaj and Antonetti, 2004; Balda and Matter, 2009). Since ZO-1 is not a direct target of miR-155, the exact mechanism of this regulation is unclear. Based on the analyses, miR-155 downregulation-induced ZO-1 stability could be mediated by the upregulation of a direct miR-155 target protein Rheb. Interestingly, stabilization of ZO-1 after miR-155 inhibition was also detected in human brain vascular endothelial cells *in vitro* (our unpublished data; Lopez-Ramirez et al., 2014). In these studies, the increased expression of ZO-1 and its stabilization on the endothelial cell membrane was associated with miR-155 direct target protein claudin-1. Identification of new miR-155 target proteins in the future could further clarify the effect of miR-155 activity on ZO-1 protein.

In the *in vivo* setting, other direct miR-155 targets upregulated in the inhibitor-injected mouse brain could broadly influence vascular function and brain tissue remodeling. These molecules are implicated in the regulation of angiogenesis, vascular stabilization and remodeling as well as vasodilation, neurovascular inflammation, and neuroprotection and thus could significantly contribute to the observed beneficial neurovascular effect of miR-155 inhibition (**Figure 1**). Detected elevated expression of SMAD5 protein may be associated with the activation of the bone morphogenetic protein (BMP) pathway, implicated in reduction of inflammation and vasculature stabilization (Marchuk et al., 2003; Pardali et al., 2010). Increased expression of Rictor, a major component of mTORC2 complex, could activate mTOR signaling, implicated in supporting angiogenesis (through activation of nitric oxide signaling) and neuroprotection after stroke (Karar and Maity, 2011; Chong et al., 2013). Endothelial nitric oxide synthase (eNOS) is associated with the improvement of blood flow and decreased rates of neuronal injury (Srivastava et al., 2012). Neuroprotective and overall pro-regenerative effect of miR-155 inhibition could be also mediated via its influence on brain-derived neurotrophic factor (BDNF) expression (Varendi et al., 2014).

In addition to these possible mechanisms, downregulation of pro-inflammatory miR-155 could improve the recovery outcome by significantly influencing post-stroke inflammation. The effect of miR-155 silencing was characterized by a suppression of an early, transient, harmful increase of pro-inflammatory cytokines, followed by sustained upregulation of neuroprotective cytokines at the later stages of stroke recovery (14 days after stroke). At 7 days after dMCAO, in the anti-miR-155 injected animals, there was a decreased expression of pro-inflammatory cytokines CCL12 and CXCL3, implicated in vascular inflammation, accompanied by the upregulation of miR-155 direct targets and cytokine suppressors SHIP-1 and SOCS-1 as well as downregulation of JAK/STAT (Janus kinase/signal transducers and activators of transcription) pathway-mediated cytokine signaling. miR-155 inhibition resulted in a reduced number of phagocytic perivascular microglia/macrophages (M/Ms) and decreased expression of macrophage marker CD45 and active phagocytosis marker CD68 (Pena-Philippides et al., 2016). Based on these findings, we concluded that miR-155 induced repression of cytokine signaling and decreased M/M phagocytic activity could contribute to preservation of TJs observed at 7 days after dMCAO.

At 14 days after dMCAO there was a sustained increase in expression of IL-10 in the inhibitor-injected animals. This major anti-inflammatory cytokine was shown to trigger an anti-inflammatory response beneficial for stroke outcome (Bazzoni et al., 2010; Perez-de Puig et al., 2013). High levels of IL-10 after the miR-155 inhibition could be induced by the observed prolonged elevation of miR-155 target and transcription factor C/EBP-β. Among other cytokines, with the increased expression at 14 days, were Il-4, IL-5, IL-6, and Il-17, which are characterized by context-dependent dual action and have a significant impact on neuroprotection and overall stroke outcome (Kim et al., 1995; Loddick et al., 1998; Li et al., 2001; Jiang et al., 2008; Erta et al., 2012). In contrast to 7 days, at 14 days after stroke there was an increase of CD45 and CD68-positive M/Ms in the peri-infarct area of miR-155 inhibited mice (Pena-Philippides et al., 2016). According to the literature, Iba-1/CD45-positive macrophages expressing active phagocytosis marker CD68 facilitate the brain recovery process following stroke (Wattananit et al., 2016). Based on our data, we propose that miR-155 inhibition at 48 h after stroke results in suppression of early transient harmful actions of the activated M/Ms at 7 days, followed by an enhancement of their protective and reparative functions at 14 days after dMCAO.

## Conclusion

Based on these findings, I conclude that systemic inhibition of miR-155 at 48 h following the experimental cerebral ischemia supports cerebral microvasculature and improves cerebral blood supply to the peri-infarct area of stroke at 7 days after stroke. These constructive processes following miR-155 downregulation are achieved via direct preservation of TJ integrity and suppression of the early stage post-stroke inflammation. The vascular support at 7 days results in neuroprotection, reduction of the infarct size, and improvement in functional recovery at the later stages (21 days) of regeneration. This recovery mechanism is facilitated by: (1) the initial preservation of vascular integrity, which prevents the propagation of the ischemic damage into the peri-infarct area; (2) the activation of IL-10-mediated neuroprotective mechanisms, and (3) transition from harmful phenotype toward the neuroprotective and reparative microglia/macrophage phenotype. All these support mechanisms could be mediated via the activation of miR-155 direct target proteins, including Rheb, SMAD5, Rictor, eNOS, SOCS-1, SHIP-1, and C/EBP-β.

## Challenges and Future Implications

As we observed in our studies, silencing of a single miRNA can regulate a broad set of target genes and trigger synergistic therapeutic effect. Based on this advantage, *in vivo* regulation of miRNAs after stroke could become a promising approach to cerebral regeneration. One of the major challenges of miRNA-based therapy in patients is achieving specific, safe, and efficient regulation of particular miRNA expression. Systemic delivery of miRNA inhibitors and mimics is problematic because of their instability in blood circulation. Therefore, lipid-based delivery vehicles, viral vectors, and nanoparticle-conjugated oligonucleotides are utilized for the introduction of synthetic miRNA inhibitors and mimics *in vivo*. Recently developed Locked Nucleic Acid (LNA)-based technology (successfully used in our *in vivo* studies) greatly increases the affinity of the inhibitors for their target microRNAs, improves their resistance to enzymatic degradation, and minimizes off-target effects. Future innovations around the delivery techniques are expected, as miRNA-based treatment remains to be of great interest for the pharmacological industry. Delivery of the antisense inhibitors across the blood–brain barrier creates an additional challenge. This problem is minimized due to increased BBB leakage following stroke; however, impaired cerebral circulation could affect the inhibition efficacy.

Discovery of more stroke-associated miRNAs could lead to the development of a combinatory therapy involving the regulation of multiple miRNA expression: targeting a subset of genes with multiple miRNAs should enhance the therapeutic effect. Certain caution should be given to possible side effects of these molecules in human trials, with specific emphasis on safety, tolerability, and efficacy of treatment. In fact, systemic miR-155 inhibition may influence immune function and negatively interfere with post-stroke recovery in humans. The optimal dose and time of the therapeutic intervention with specific microRNA inhibitors or mimics should be carefully determined to avoid any destructive intervention into the natural regeneration process. This therapeutic approach should be based on the knowledge of specific microRNA function, including its influence on the viability, proliferation, and differentiation of the brain tissue components as well as its possible effect on cerebral vasculature and blood flow, post-stroke inflammation, thrombosis, and atherosclerosis, etc. The degree of the inhibition or overexpression of specific miRNAs should also be taken into consideration; moderate systemic downregulation or upregulation could be a better choice to avoid secondary non-specific off-target effects or negative side effects such as impairment of the immune function.

## Author Contributions

The author confirms being the sole contributor of this work and approved it for publication.

## Conflict of Interest Statement

The author declares that the research was conducted in the absence of any commercial or financial relationships that could be construed as a potential conflict of interest.
